# Seasonal peaks and risk factors of respiratory syncytial virus infections related hospitalization of preterm infants in Taiwan

**DOI:** 10.1371/journal.pone.0197410

**Published:** 2018-05-10

**Authors:** Hsin Chi, Ching-Hu Chung, Yuh-Jyh Lin, Chyi-Her Lin

**Affiliations:** 1 Department of Pediatrics, Mackay Memorial Hospital and Mackay Children’s Hospital, Taipei, Taiwan; 2 Department of Medicine, Mackay Medical College, New Taipei City, Taiwan; 3 Department of Pediatrics, National Cheng Kung University Hospital and College of Medicine, National Cheng Kung University, Tainan, Taiwan; Chang Gung Memorial Hospital, TAIWAN

## Abstract

**Objectives:**

To assess the nationwide seasonal peaks, risk factors, and utilization of medical resources of respiratory syncytial virus-associated hospitalization (RSVH) in preterm infants in Taiwan.

**Study design:**

A Taiwan nationwide birth cohort was extracted from the Birth Certificate Application Database during 2007–2009 and prospectively linked to the National Health Insurance database. We evaluated the seasonal peaks and risk factors (gestational age [GA], chronologic age [CA], and bronchopulmonary dysplasia [BPD]) associated with the RSVH of preterm infants. The length of hospital stays (LOS), care in intensive care unit (ICU), and use of mechanical ventilation (MV) were also analyzed.

**Results:**

There is a total duration of 9 months of RSVH season in Taiwan, three seasonal peaks and two seasonal peaks of RSVH in preterm infants with BPD and without BPD, respectively. Preterm infants had significantly higher RSVH rate than term infants (2.6% vs 0.9%, *p*<0.0001). Preterm infants born at 29–35 weeks of gestational age (wGA) with BPD had significantly higher RSVH rate than those without BPD (*p*<0.0001). Preterm infants without BPD born at < 32 wGA had higher RSVH rate than those born at 33–35 wGA (*p*<0.0001). Overall, 56.4% of RSVH occurred within 9 months of CA. Preterm infants with BPD had significantly higher ICU admission rate within 18 months of CA (*p*<0.0001), MV usage within 12 months of CA (*p*<0.0001) and LOS within 18 months of CA (*p*<0.001) than those without BPD. RSVH occurred within 6 months of CA was significantly associated with higher ICU admission rate (*p*<0.0001), MV usage (*p* = 0.0002) and longer LOS (*p*<0.001) in preterm infants without BPD.

**Conclusions:**

There is a total duration of 9 months of RSVH season in Taiwan. Preterm < 32 wGA, BPD, and CA within 6 months were risk factors of RSVH which also contribute to higher utilization of medical resources.

## Introduction

Respiratory syncytial virus (RSV) is an important virus for lower respiratory tract infections among infants and young children [1,2]. Nearly all children have been infected at least once by 2 years of age, with 1–2% requiring hospitalization [3,4]. Populations at risk for RSV-associated hospitalization (RSVH) include infants who are born before 35 weeks of gestational age (wGA), with bronchopulmonary dysplasia (BPD), and who have hemodynamically significant congenital heart disease (HS-CHD) [5]. Chronologic age (CA) is one of the most important risk factor for RSVH and most of pediatric RSVH occur in the first 5 months after birth [5,6]. Since 1998, the American Academy of Pediatrics (AAP) has recommended palivizumab prophylaxis guidelines against RSV according to their BPD status, gestational age(GA) and CA at the beginning of the RSV season to prevent the respiratory complication [7]. Because of the high costs of RSV prophylaxis [8] and additional data regarding seasonality of RSV infection, AAP revised its policy and restricted the palivizumab prophylaxis to preterm infants who were born before 32 wGA and infants born at 32 to 35 wGA with certain risks in 2009 [9]. In 2014, the AAP further evolved palivizumab prophylaxis to preterm infants born before 29 wGA, born at <32 weeks with BPD of prematurity, and infants with HS-CHD [10].

Prevention of RSV is complicated by considerable variation in RSV seasonality across geographic locations [11]. The current AAP guideline recommends a series of 5 monthly injections which simply reflects the average length of the RSV season across the United States, Canada, and the United Kingdom without considering the variation in regional seasonality [12]. The RSV infection has been reported to be ongoing throughout the year in countries with warmer climates [13,14].

In our previous hospital-based study, preterm infants are most susceptible to experience RSVH before 9 months of CA and there is an extended RSV season for 10 months in northern Taiwan [15]. The results implied that the AAP regimen may not be appropriately applicable. A nationwide study to better understand the seasonal peaks of RSV infection could provide evidence for policy establishment of RSV prophylaxis guidelines in Taiwan as well as other subtropical countries. The purposes of this study were to retrieve and analyze the database to determine the seasonal peaks of RSVH and risk factors with high medical expenditures and resource needs, with focus on preterm birth and BPD in the pre-palivizumab era in Taiwan.

## Materials and methods

### Research ethics approval

All the data used in the present study were anonymous without any identifiable personal information and were available through formal application to the Health and Welfare Data Science Center at Ministry of Health and Welfare, Taiwan. The protocol of this study was approved by the Institution Review Board of Buddhist Tzu Chi General Hospital Research Ethics Committee (Protocol Number: IRB 101–42) (S1 Fig).

### Data source

The Taiwan National Health Insurance (NHI) claims database comprises complete outpatient visits, hospital admissions, prescriptions, disease, and vital status for the 23 million people in Taiwan. We established the longitudinal medical history of each beneficiary by linking electronic administrative and claims datasets. The GA of newborns was identified from the Birth Certificate Application Database through the civil identification number unique to each beneficiary and date of birth.

### Study population

We examined the Birth Certificate Application Database for the source population between 2007–2009 to identify all newborns at different GA when the policy of palivizumab prophylaxis was not implemented in Taiwan. In this analysis, preterm infants with BPD were categorized separately to reduce potential confounding effects. Infants were classified as preterm (<29, 29–32, and 33–35 wGA) and term infants (≥37 wGA) based on diagnostic coding. These infants were followed until Dec. 2011. All participants were observed for at least two years after birth. BPD was defined as preterm infants less than 35 wGA who required oxygen therapy at 36 weeks of post-conceptual age and was identified by The International Classification of Diseases, 9th Revision, Clinical Modification (ICD-9-CM) code 7707 (chronic respiratory disease arising in the perinatal period). Patients were classified as having RSVH if they had one hospital admission with one of the diagnoses at discharge: ICD-9-CM code 079.6 (RSV infection), 480.1 (RSV pneumonia), and 466.11 (RSV bronchiolitis).

#### Monthly incidence of RSVH

Monthly RSVH rate was calculated by monthly RSVH number divided by the number of each group of preterm infants aged 2 years or younger. The average RSVH rate was calculated by total number of RSVH divided by the number of each group of preterm infants aged 2 years or younger. The months with RSVH rates lower than the average rate in each group were defined as ‘‘non-epidemic months”. The mean monthly RSVH rate of ‘‘non-epidemic months” was defined as the baseline rate. The RSV season was defined as RSVH rate in two or more consecutive months that was above the baseline rate.

#### Outcome measurement

CA at the time of RSVH was measured using the birth date and admission date. Children with RSVH were followed every three months (0–3, 4–6, 7–9, 10–12, 13–15, 16–18, 19–21 and 22–24 month). The severity of RSVH was measured by whether they needed the assistance of mechanical ventilation (MV) and admission to intensive care unit (ICU) during hospitalization. The utilization of MV was identified by ICD-9-CM procedure code 96.7x (other continuous invasive mechanical ventilation). The other outcome variables included length of hospital stay (LOS), recorded by the total number of days of RSVH per visit.

### Data analyses

The risk factors for RSVH included the GA, BPD, and CA. We performed the trend tests to examine linear gradient relationship with the risk of endpoints of interest. We also used the chi-square test to examine the difference in hospitalization rates among the 5 groups, including the BPD group, three GA groups (<29, 29–32 and 33–35 wGA) and term infants group. All statistical analyses were performed using SAS, version 9.1 (SAS Institute, Cary, NC). Effect estimates were presented as odds ratios (OR), with 95% confidence intervals (95% CI). A *p* value of less than 0.05 was considered statistically significant.

## Results

### Study population

**Fig 1:**
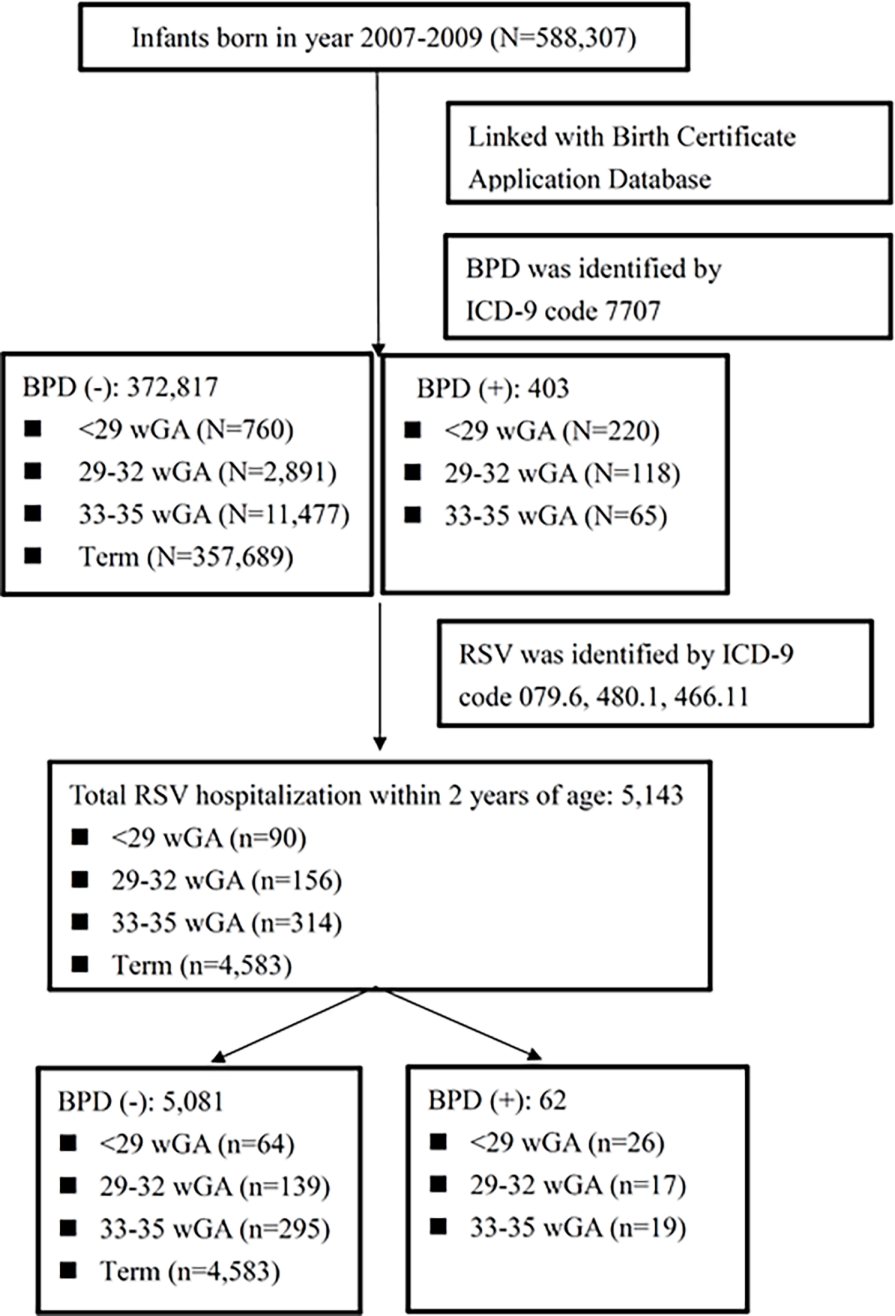
Flow chart of children aged 2 years or younger included in the study. BPD, bronchopulmonary dysplasia; RSV, Respiratory syncytial virus; wGA, week of gestational age; ICD, International Statistical Classification of Diseases.

From January 2007 to December 2009, 588,307 infants were born in Taiwan. Of whom 373,220 infants were linked between Birth Certificate and NHI database (Fig 1). We identified 980 preterm infants born at <29 wGA (220 with BPD), 3,009 between 29–32 wGA (118 with BPD), and 11,542 between 33–35 wGA (65 with BPD). We also identified 357,689 term infants as the control group. Among the control group, 3,236 (0.9%) and 1,347 (0.4%) had RSVH during the first and second year of life. The overall rate of RSVH in preterm infants was 2.6% (400/15,531) in the first year and 1.1% (160/15,388) in the second year. Both were significantly higher than that in term infants in the first year (OR 2.9, 95% CI 2.6–3.2, *p* <0.0001) and in the second year (OR 3.0, 95% CI 3.5–3.4, *p* <0.0001).

#### Monthly incidence of RSVH

**Fig 2:**
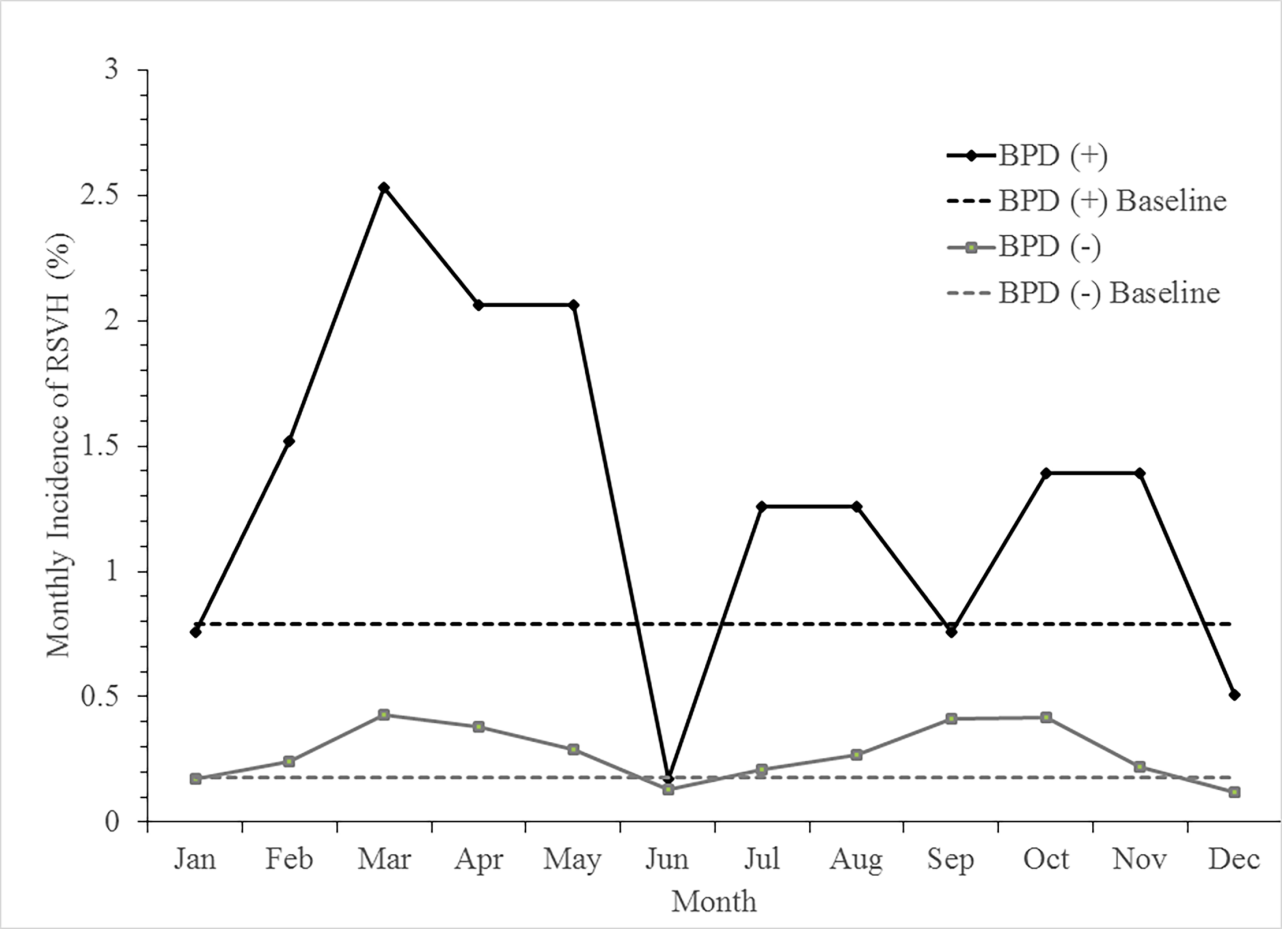
Monthly incidence of RSVH of preterm infants in the first 2 years of age. The baseline monthly incidence of RSVH was 0.79% of preterm children with BPD and 0.18% of infants without BPD. BPD, bronchopulmonary dysplasia; RSVH, Respiratory syncytial virus-associated hospitalization.

Fig 2 shows the monthly distribution of the RSVH during the first 2 years of age. The average monthly incidence of RSVH was 1.3% and 0.3% of preterm infants with BPD and without BPD, respectively. The RSV seasons of infants with BPD were in February–May, July–August and October–November. The RSV seasons of infants without BPD were in February–May and July–November. The monthly distribution of RSVH rates revealed three RSV seasonal peaks of preterm infants with BPD and two RSV seasonal peaks of preterm infants without BPD. Overall, a total duration of 9 months of RSV season in Taiwan.

#### Risk factors for RSVH of preterm infants

Table 1 summarizes comparisons of preterm infants with RSVH by GA and BPD status in the first 2 years. Compared to term infants, preterm infants born at < 29 wGA, 29–32 wGA, and 33–35 wGA had a significantly higher rate of RSVH in the first year and second year of life. The preterm infants born at < 29 wGA and 29–32 wGA had significantly higher rate of RSVH than those born at 33–35 wGA. There was no significant difference in the RSVH rates between preterm infants born at <29 wGA and 29–32 wGA in the first year (OR 1.4, 95% CI 0.9–2.0, *p* = 0.08). However, infants born at <29 wGA had higher RSVH rate than those born at 29–32 wGA in the second year (OR 2.9, 95% CI 1.8–4.9, *p*<0.0001). Regardless of the GA, preterm infants with BPD had higher RSVH rate than those without BPD in the first year (10.9% vs 2.4%, OR 5.0, 95% CI 3.6–7.0, *p* <0.0001) and second year (5.1% vs 0.9%, OR 5.6, 95% CI 3.5–9.2, *p* <0.0001). Of the infants born at 29–32 wGA without BPD, those with BPD were also more likely to have RSVH in the first year (9.3% vs 3.6%, OR 2.8, 95% CI 1.5–5.2, *p* = 0.0015) and second year (5.4% vs 1.2%, OR 4.5, 95% CI 2.0–10.2, *p* = 0.0002). Similarly, BPD was associated with higher RSVH rate among the preterm infants born at 33–35 wGA in the first year (21.5% vs 1.9%, OR 14.4, 95% CI 9.1–22.8, *p* <0.0001) and second year (8.9% vs 0.7%, OR 13.9, 95% CI 6.8–28.7, *p* <0.0001). However, there was no association between BPD and RSVH among preterm infants born <29 wGA.

**Table 1:** The comparisons of RSVH rates among each GA groups and BPD status of preterm infants in the first 2 years of age.

Gestational age	<29 wGA		29–32 wGA		33–35 wGA	
	1^st^ Year	2^nd^ Year	1^st^ Year	2^nd^ Year	1^st^ Year	2^nd^ Year
Overall, % (n/N)	5.7 (56/980)	3.6 (34/942)	3.8 (115/3,009)	1.7 (50/2,957)	2.2 (229/11,542)	0.7 (85/11,487)
No BPD, % (n/N)	5.0 (38/767)	3.5 (26/736)	3.6 (104/2,891)	1.2 (35/2,845)	1.9 (215/11,477)	0.7 (80/11,431)
OR (95% CI) vs Term	5.7 (4.1–7.9)*	9.7 (6.5–14.3)*	4.1 (3.4–5.0)*	3.3 (2.3–4.6)*	2.1 (1.8–2.4)*	1.9 (1.5–2.3)*
OR (95% CI) vs 33–35 wGA	2.7 (1.9–3.9)*	5.2 (3.3–8.1)*	2.0 (1.5–2.5)*	1.8 (1.2–2.6)^‡^	**–**	**–**
OR (95% CI) vs 29–32 wGA	1.4 (0.9–2.0)	2.9 (1.8–4.9)*	–	–	**–**	**–**
BPD, % (n/N)	8.5 (18/213)	3.9 (8/206)	9.3 (11/118)	5.4 (6/112)	21.5 (14/65)	8.9 (5/56)
OR (95% CI) vs no BPD	1.8 (0.9–3.7)	1.1 (0.5–2.5)	2.8 (1.5–5.2)^‡^	4.5 (2.0–10.2)^‡^	14.4 (9.1–22.8)*	13.9 (6.8–28.7)*

BPD, bronchopulmonary dysplasia; n, number of RSVH by each GA group; N, number of preterm infants; OR, odds ratio; 95% CI, 95% confidence intervals; wGA: weeks of gestational age

^†^*p*<0.05

^‡^*p*<0.01

^§^*p*<0.001, and

*p<0.0001 indicate comparisons between each other in category of GA and BPD.

#### Rate of RSVH of preterm infants by GA, BPD, and CA

**Fig 3:**
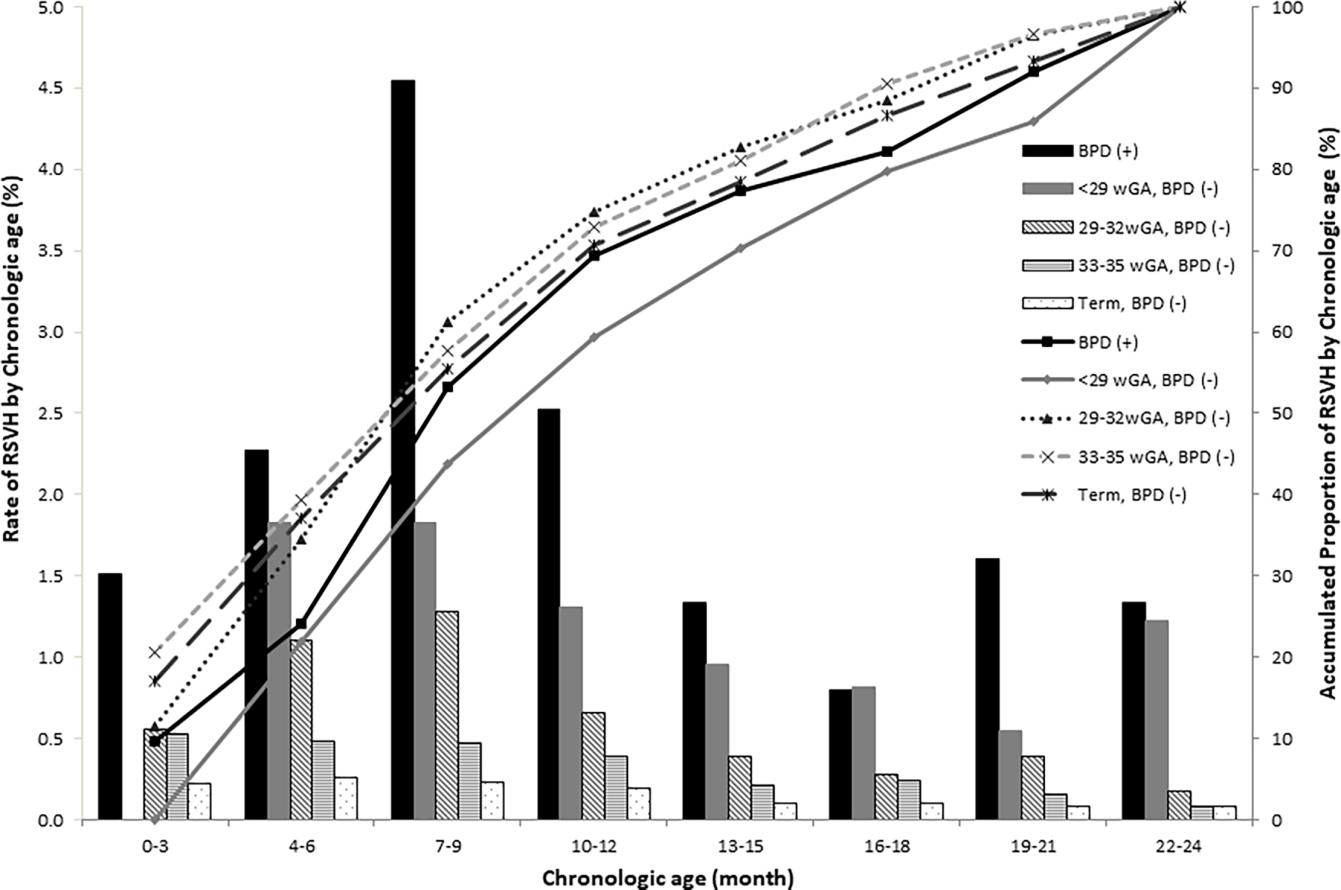
The rate and proportion of RSVH by CA of onset categorized by GA and BPD status. No RSVH occurred within 3 months of CA in infants born at <29 wGA without BPD. The incidence of RSVH started to increase after 3 months and peaked between 7–9 months of CA. Among preterm infants, 56.4% of RSVH occurred within first 9 months of CA, 71.4% within 12 months, and 87.9% within 18 months. BPD, bronchopulmonary dysplasia; CA, chronologic age; GA, gestational age; RSVH, Respiratory syncytial virus associated hospitalization; wGA, week of gestational age.

Fig 3 summarizes the rate and proportion of children hospitalized due to RSV infection by CA of onset (categorized by GA and BPD status). Overall, 56.4% of RSVH occurred within first 9 months of CA, 71.4% occurred within 12 months, and 87.9% occurred within 18 months. No RSVH occurred within 3 months of CA in infants born at <29 wGA without BPD. The incidence of RSVH started to increase after 3 months and peaked between 7–9 months of CA in all groups, irrespective of the presence of BPD. Of the infants with BPD, RSVH occurred mostly in 7–9 months of CA, followed by 10–12 months and then 4–6 months of CA.

#### Trends of admission to ICU, use of MV and LOS in RSVH

**Fig 4:**
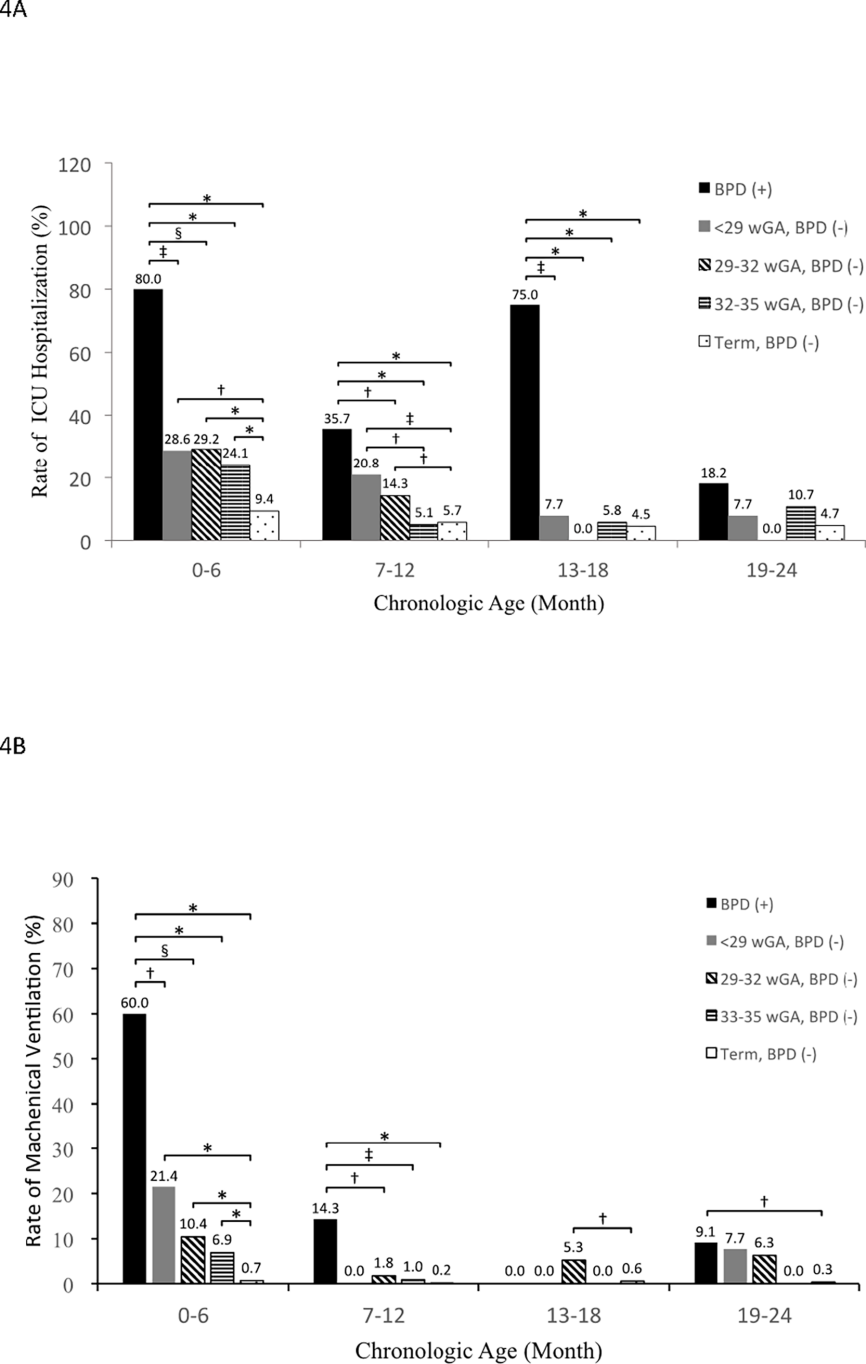
Rate of ICU admission and MV usage of RSVH by GA and CA in first 2 years of age. (A). Higher rate of ICU admission was associated with preterm infants with BPD within 18 months of CA and without BPD within 12 months of CA. (B). MV utilization was more frequent among preterm infants with BPD within 12 months of CA. BPD, bronchopulmonary dysplasia; CA, chronologic age; GA, gestational age; ICU, intensive care unit; MV, Mechanical Ventilation; RSVH, Respiratory syncytial virus associated hospitalization; wGA, week of gestational age. ^†^*p*<0.05, ^‡^*p*<0.01, ^§^*p*<0.001, and *p<0.0001 indicate comparisons between indicated groups in each category of CA.

The overall ICU admission rate during RSVH was significantly higher in preterm infants than term children (18.2% vs 6.7%, OR 3.1, 95% CI 2.4–3.9, *p*<0.0001). The preterm infants with BPD had significantly higher ICU admission rate than those without BPD (48.4% vs 14.5%, OR 5.6, 95% CI 3.3–9.3, *p*<0.0001). ICU admission rate was reduced as the CA increased (Fig 4A). RSVH occurred before 6 months of CA was associated with higher ICU admission than 7–24 months of CA among both preterm infants with BPD (80.0% vs 38.3%, OR 6.4, 95% CI 1.8–23.6, *p* = 0.005) and those without BPD (25.8% vs 8.1%, OR 3.9, 95% CI 2.4–6.5, *p*<0.0001).

The overall MV utilization rate during RSVH was significantly higher in preterm infants than term infants (6.3% vs 0.4%, OR 18.1, 95% CI 11.8–27.8, *p*<0.0001). In addition, the MV utilization was significantly more frequent among preterm infants with BPD than those without BPD (22.6% vs 4.2%, OR 6.6, 95% CI 3.4–12.8, *p*<0.0001) (Fig 4B). The effect of BPD on MV utilization was consistent in the first 12 months of CA (30.2% vs 5.0%, OR 8.2, 95% CI 4.0–16.5, *p*<0.0001), however, the finding was not observed in 13–24 months of CA (5.3% vs 2.1%, OR 2.6, 95% CI 0.3–28.0, *p* = 0.41). Among the preterm infants without BPD, the MV utilization rate was significantly higher within 6 months of CA than 7–24 months of CA (9.0% vs 1.6%, OR 6.2, 95% CI 2.5–15.4, *p* = 0.0002). Among preterm infants without BPD, MV utilization rate was not affected by GA in the same CA category.

The mean length of stay (LOS) among preterm infants without BPD ranged from 4.7–10.3 days, and that among those with BPD ranged from 5.9–12.5 days (Table 2). Preterm infants with BPD had longer LOS than term infants without BPD if the RSVH occurred within 18 months of CA. Preterm infants born at <29 wGA without BPD had longer LOS than term infants without BPD if the RSVH occurred within 18 months of CA. The RSVH within 6 months of CA was associated with longer LOS than term infants without BPD of all GA groups without BPD.

**Table 2:** The LOS of RSVH by GA and with or without BPD categorized by CA.

Chronologic Age (month)	0–6	7–12	13–18	19–24
BPD	10.9 ± 2.1^§^	12.2 ± 3.7^§^	12.5 ± 4.6^§^	5.9 ± 1.3
<29 wGA, no BPD	9.3 ± 3.7^§^	7.2 ± 2.48^‡^	9.7 ± 4.1^§^	6.3 ± 2.2
29–32 wGA, no BPD	10.3 ± 3.9^§^	6.0 ± 2.4	4.7 ± 1.9	4.7 ± 2.9
33–35 wGA, no BPD	8.7 ± 1.5^§^	5.9 ± 2.0	5. 5± 2.3	8.1 ± 3.0 ^§^
Term, no BPD (reference)	6.7 ± 2.0	5.6 ± 1.7	5.1 ± 1.5	5.1 ± 2.0

BPD, bronchopulmonary dysplasia; CA, chronologic age; GA, gestational age; ICU, intensive care unit; LOS, length of hospital stays; MV, Mechanical Ventilation; RSVH, Respiratory syncytial virus associated hospitalization; wGA, week of gestational age

^†^*p*<0.05

^‡^*p*<0.01

^§^*p*<0.001, and

*p<0.0001 indicate comparisons between Term, no BPD (reference) and each other group in each category of chronologic age.

## Discussion

This study is the first nationwide birth cohort analysis of RSVH among preterm infants in Taiwan. There is a total duration of 9 months of RSVH season of preterm infants. More than half of the RSVH occurred within 9 months of CA. We found that preterm, younger CA, and BPD were the risk factors for RSVH. Preterm infants with BPD had higher rate of RSVH, ICU admission, MV use, and longer LOS within 12 months of CA. RSVH occurring within 6 months of CA in preterm infants was associated with a higher ICU admission rate, MV usage and longer LOS. Our study showed the overall RSVH rate was 2.6%; 8.5% for preterm infants with BPD, 5.7% for born at <28 wGA, 3.8% for born at 29–32 wGA, 2.2% for born at 33–35 wGA and 0.9% for term infants in the first year. In the United Kingdom, a research using the Hospital Episode Statistics database reveals that the RSVH rate was 47.3 per 1000 of preterm infants and was 22,4 per 1000 of term infants in the first year [16]. In an Australia study between 2001 and 2010, the population-based incidence of RSVH (per 1000 child-years) was 81.5 for children with BPD, 39.0 for children born at <28 wGA, 27.0 for children born at 28–31 wGA, and 10.2 for preterm children born at 32–36 wGA [17]. Our RSVH rate is relatively lower than the reports in the United Kingdom and it might be due to the differences in the climate and humidity between subtropical and temperate regions resulted from disparate latitudes [18]. However, the RSVH rate in our study is still comparable with Australia study.

Taiwan is a subtropical country (latitude 23°0'N~25°5'N), and there is no dominant RSV season like countries in the temperate zones. The two seasonal peaks of RSVH in our study is consistent with that of the previous studies in Taiwan [19,20]. The RSV infection has been reported to be ongoing throughout the year in countries with warmer climates [13,14], as well as among preterm infants in a subtropical climate [15]. The 5 monthly injections of palivizumab at 15 mg/kg per dose is proposed to provide around 6 months of desired serum palivizumab concentrations for the high-risk infants [21]. However, based on our findings of prolonged RSV seasonality, the guideline on RSV prophylaxis with palivizumab by AAP may not be suitable in Taiwan.

It is challenging to choose an appropriate cutoff of gestational age for which palivizumab prophylaxis may be considered for preterm infants without other indications. Data consistently demonstrates the greatest risk for RSVH is among preterm infants born before 29 wGA, who have 2 to 4 times the RSVH rates compared to those born at later GA [5,6,22]. Prematurity is the only factor that significantly increased the risk of being hospitalized for bronchiolitis [23,24]. A Tennessee study showed that the RSVH incidence is directly correlated with lower GA, indicating that prematurity *per se* is associated with increased severity of RSV infections [5]. Preterm infants have poorer immunity against RSV infection due to immature respiratory and immune systems [25]. It is assumed that prematurity represents a major risk factor for RSVH, due to low levels of transplacental transfer of RSV neutralizing maternal antibodies [26,27]. Two studies analyzing data from 1999–2004 and 2005–2011 demonstrated a 2 to 3 folds increase in risk of RSVH among infants born between 32–34 wGA when compared to full-term infants [28,29]. In our study, we also found that preterm infants born at lower GA had higher risk of RSVH than full term infants.

The rate of RSVH in preterm infants with BPD is higher than those without BPD [15,24]. Boyce *et al*. suggested that BPD is a risk factor for RSVH and has remained until 2 years of age in the Tennessee Medicaid Program [5]. We also found that preterm infants with BPD were at least two times more likely to have RSVH than infants without BPD within the first two years. From the grouped cohort study by Weisman *et al* [26], the weighted mean RSVH incidence for infants with BPD is 17% in the first 2 years of life. We found that preterm infants with BPD also had higher rate of RSVH. Our findings are also consistent with the IRIS study [30], which reports that preterm infants either born at ≤32 wGA or with BPD are at risk of RSVH.

In temperate climate countries, the RSV season is confined in late fall or winter, and approximately 50% of RSVH occur among infants younger than 6 months [31,32]. By contrast, this study revealed that more than half of the RSVH occurred within 9 months of CA in preterm infants born <29 wGA, 29–32 wGA and preterm infants with BPD regardless GA. No RSVH occurred within 3 months of CA among preterm infants <29 wGA without BPD. It is likely that these infants are still in the hospitals in the first 2–3 months of CA and have less chance of exposure to RSV virus.

The need for ICU care and MV are particularly important outcomes of RSVH because these are indicators of the severity of diseases. The percentage of admission to ICU ranges from 13.3–60.7% in RSVH infants born at 29–35 wGA [33]. Premature birth and underlying medical conditions increase the risk of hospitalization and are associated with severe clinical manifestations, such as more frequent requirement for MV, ICU admission, longer LOS, and increased mortality [34,35]. In our study, we found the highest rate of ICU care and MV requirement in RSVH was among infants within 6 months of CA. The rate of ICU care was similar in preterm infants who was born at <29 wGA and 29–32 wGA. These results indicated that earlier RSV infection and younger preterm infants were associated with increased illness severity. An observational study of RSVH among infants born between 29–32 wGA and < 3 months of age showed that 68% of the infants require ICU admission and 44% require MV [36]. The severity of RSVH is strongly associated with prolonged LOS [37]. Paralleling to the MV and ICU usage, the infants with BPD and their RSVH occurs earlier than 6 months of CA had longer LOS in our study.

The major limitation is that this study focused mainly on reported data, and the true rate of RSVH and BPD might be underrated, because they might not be recognized or corded at discharge. Most probably, only the severe bronchiolitis was test for the possibility of RSV infection. Secondly, the milder cases of BPD might tend to be missed more frequently than more severe cases in our study. According to the validation study of ICD-9 diagnostic codes of BPD in Canada [38], the specificity of the use of ICD-9 diagnostic codes for BPD in the Quebec provincial health care databases is acceptable to allow its use and milder cases will likely result in an underestimation of the impacts of BPD. We need prospective investigation to monitor the epidemiological patterns of RSV infections in the community, and collaborating local clinics, regional hospitals, and medical centers will provide more details regarding RSV infections.

## Conclusion

There are two seasonal peaks of RSVH in Taiwan. Preterm infants without BPD born at< 32 wGA had higher RSVH rate. BPD and CA within 6 months were risk factors of RSVH which also contribute to higher utilization of medical resources. The current Taiwanese recommendations for RSV prophylaxis specify six doses of palivizumab, targeting preterm infants born before 30^6/7^ wGA or those born before 35^6/7^ wGA with BPD. According to our results, the palivizumab prophylaxis in preterm infants with BPD might be extended to 12 months of CA to prevent the severer RSVH. However, further prospective study is required.

## Supporting information

S1 Fig Certification of Institution Review Board. (JPG)
