# Supplementary figures and images for: Seasonal peaks and risk factors of respiratory syncytial virus infections related hospitalization of preterm infants in Taiwan
Source: PLoS One. 2018 May 10;13(5):e0197410. doi: 10.1371/journal.pone.0197410 (PMC5944988; doi:10.1371/journal.pone.0197410)

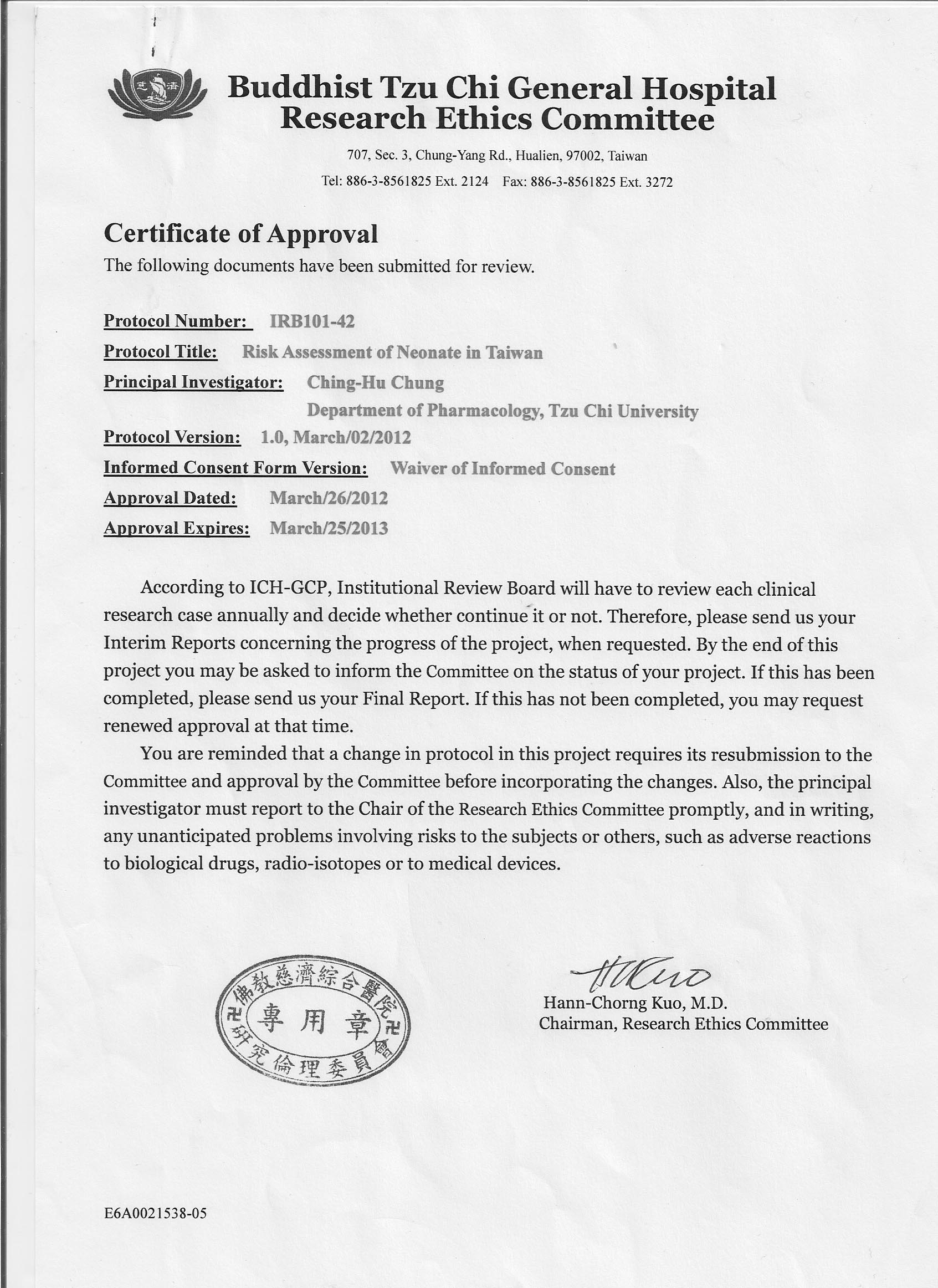

Supplement: S1 Fig — (JPG) [file pone.0197410.s001.jpg]
